# „S.P.A.S.S.“ – eine „Systemic Personal Assistance Service Solution“ zur Unterstützung von Mitarbeiter:innen zwischen sozialer Einsamkeit und Überforderung

**DOI:** 10.1007/s11613-022-00788-x

**Published:** 2022-10-18

**Authors:** Franz Schiermayr, Charlotte Sweet

**Affiliations:** Fakultät für Medizintechnik und Angewandte Sozialwissenschaften, FH OÖ Studienbetriebs GmbH, Garnisonstraße 21, 4020 Linz, Österreich

**Keywords:** Homeoffice, Psychische Belastung, Systemisches Coaching-Tool, Home office, Mental stress, Systemic tool

## Abstract

Das beschriebene Projekt fokussiert Auslassungen der Digitalisierung insbesondere dort, wo Mitarbeitende vorwiegend im Homeoffice arbeiten. Es wurden die bereits bekannten Belastungsfaktoren bei der Nutzung digitaler Medien in einer qualitativen Erhebung bestätigt und um individuelle sowie Covid-19 bedingte Aspekte erweitert. Um den Einschränkungen durch die digitale Systematik konstruktiv entgegenzuwirken, wurde ein flexibel anwendbarer S.P.A.S.S. (Systemic Personal Assistance Service Solution) Workshop als Tool entwickelt. Dieses Coaching-Tool fördert betriebliche Kommunikation sowie die Selbstorganisation und Reflexion der Mitarbeitenden zur konstruktiven Gestaltung von Arbeit.

## Abstract (en)

The presented project focuses on omissions as part of digitization, particularly where employees are working primarily in home offices. The previously identified stress factors arising from the use of digital media were confirmed in a qualitative survey and expanded to include individual, as well as Covid-19 related aspects. In order to constructively counteract the limitations caused by the digital system, a flexibly applicable S.P.A.S.S. (Systemic Personal Assistance Service Solution) workshop was developed as a coaching tool. This coaching tool promotes internal communication, as well as the self-organization and reflection skills of employees.

## Einleitung

In vielen Bereichen der Arbeitswelt verändern sich durch Digitalisierung, Virtualisierung und die Einbindung von künstlichen Intelligenzen die Anforderungen an Arbeitnehmer:innen und somit auch die Stressfaktoren, die langfristig den Arbeitsprozess belasten. Studien und Praxis belegen, dass die Umstellung auf vorwiegend digitale Datenverarbeitung und Kommunikation in Organisationen für Mitarbeiter:innen neue Stressfaktoren birgt, die langfristig ihre psychische Belastung deutlich erhöhen (Knieps und Pfaff 2017). In der sogenannten „Corona-Krise“ befanden sich viele Mitarbeiter:innen ausschließlich im Homeoffice und waren aufgefordert, ihre Arbeit in einem völlig anderen Kontext selbstverantwortlich zu erledigen. Die Ergebnisse verschiedenster Studien (Ernst 2020; Göpner-Reinecke 2019; Ahlers 2021) und die Beobachtungen von Personalverantwortlichen, Betriebsrät:innen und Mitarbeiter:innen in dieser „Ausnahmesituation“ hinsichtlich Belastung und deren Beanspruchungsfolgen werden auch durch langjährige Erfahrungen der Projektleitung im Coaching von Mitarbeiter:innengruppen in verschiedensten Unternehmen in der Praxis bestätigt. Auch Hagemann (2018, S. 310) beschreibt, dass Belastungen im Zuge der Digitalisierung nur dann zu gefährdenden Beanspruchungen führen, wenn diese längerfristig mit Unsicherheiten oder Ängsten und Frustrationen in Verbindung stehen. „Digitale Systeme fördern […] Exkommunikation, indem sie auf willkürlichen Annahmen basierende binäre Codes verwenden und dadurch gleichberechtigte Aushandlungsprozesse verhindern“ (Sweet und Schiermayr 2020, S. 13). Diese Ängste und neuartigen Stressfaktoren des überwiegend digitalen Arbeitens sowie die Entbindung von Mitarbeiter:innen aus persönlichen und sozialen Netzwerken und Kommunikationsstrukturen stellen eine Belastung dar und können sich in der Folge als gesundheitsgefährdende Beanspruchung manifestieren.

Allerdings ist das Zusammenspiel der Belastungsfaktoren sehr komplex und lässt sich nicht mittels einfacher Zuschreibungen an die digitale Kommunikation, die individuelle persönliche Kompetenzausstattung von Mitarbeiter:innen oder als Resultat isolierter Strukturmerkmale in Unternehmen darstellen. Zwischenmenschliche Kommunikation reduziert sich nicht nur auf die Weitergabe von Information, sondern es werden Bedürfnisse, gesehen und verstanden zu werden sowie sich als zugehörig zu erleben, im persönlichen Kontakt ebenso mittransportiert. Werden nun Arbeitnehmer:innen von diesen Teilen der Kommunikation ausgeschlossen, können in der Arbeitsrealität psychische Belastung, Stress, Angst und Sinnverlust zunehmen, und die Entwicklung verschiedener Störungen wie Depression, psychosomatische Erkrankungen und Abhängigkeiten kann zusätzlich verstärkt werden (Schaff 2019, S. 316).

Auf der Basis dieser Einsicht wurde die Idee eines „Systemic Personal Assistance Program“ für Mitarbeitende, die vorwiegend im Homeoffice arbeiten, entwickelt – gefördert mit Mitteln des Zukunftsfonds Arbeit Menschen Digital der Arbeiterkammer Oberösterreich. Mitarbeitende als Teilhabende an einer ganzen Reihe komplexer sozialer Funktionssysteme sowie sich selbstorganisierender psychischer und biologischer Systeme brauchen die Erfahrung, dass sie selbstwirksam sein können, d. h. dass sie Einfluss nehmen können auf sinngerichtete Komponenten ihres Lebens und Verhaltens.

Auch wenn die Programmierung digitaler Anwendungen die Grundwerte der individuellen Interaktionen und Veränderungen vor rigide Prozessoperationen reihen, hat die Coronakrise gezeigt, dass Exkommunikation auf der Basis digitaler Umsetzung passiert – und zwar wesentlich systematischer, umfassender und effektiver, als dies ohne technologische Hilfe möglich wäre. In der vorliegenden Studie wurde deutlich, dass in der Krisenzeit durch die Umstellung auf digitale Kommunikationsmittel vor allem die Verständigung auf der vertikalen Ebene verstärkt und gestützt wurde. Mitarbeitende berichteten fast durchgängig vom guten und klaren Kontakt mit der unmittelbaren Führungsebene. Dafür gab es beträchtliche Einbußen beim horizontalen und informellen Kontakt mit Kolleg:innen auf der gleichen hierarchischen Ebene.

Grundsätzlich kann gesagt werden, dass die Digitalisierung moderner Funktionssysteme für Gesellschaften Flexibilisierung bedeutet. Dies hat Vor- und Nachteile für alle Beteiligten. Insbesondere bedeutet es, dass Regulative an die Entkopplung des Arbeitsprozesses von physischen Orten bzw. Zeitspannen angepasst werden sollten, wobei sich die Frage der Vereinbarkeit dieser Flexibilität mit Fürsorgepflichten stellt. Die Autor:innen haben auf dieser Basis theoretischer Erkenntnisse einerseits erhoben, ob die bekannten neuen Belastungsfaktoren tatsächlich mit jenen korrelieren, die Mitarbeitende in der Krisenphase kolportieren, oder ob sich durch qualitative Befragungen zusätzliche Problemfaktoren identifizieren lassen. Das daraus zu entwickelnde Workshop-Programm setzt dort an, wo die Digitalisierung ihre offensichtlichsten Grenzen offenbart – beim informellen Austausch und Dialog außerhalb hierarchischer Gegebenheiten.

## Theoretische Zugänge

Die Literatur im Themenbereich digitale Veränderungen der Arbeitswelt stellt sich sehr umfangreich dar. Eine große Anzahl von Studien zu verschiedensten Aspekten zum Thema aus der Sicht von Unternehmen und von Mitarbeiter:innen stehen zur Verfügung. Im vorliegenden Entwicklungsprojekt werden nachfolgend lediglich solche theoretischen Rahmenbedingungen dargestellt, die einen direkten Zusammenhang mit der Entwicklung des Projekts haben. Im Mittelpunkt stehen dabei die Mitarbeiter:innen und ihre Herausforderungen in ihrer Tätigkeit im Homeoffice.

### Selbstorganisation als wesentliche Anforderung im Homeoffice

Im Zuge eines zunehmenden Einsatzes von digitalen Technologien im gesellschaftlichen Alltag und damit auch in der Arbeitswelt steigen auch die Anforderungen an die Selbstorganisation bzw. das Selbstmanagement der Mitarbeitenden. Dynamik und Komplexität in der Gestaltung von sozialen und beruflichen Prozessen nimmt stetig zu. Zugleich scheint die Bedeutung von formalen Hierarchien in einzelnen Aspekten der Arbeit abzunehmen (Graf und Olbert-Bock 2019, S. 287). Der Begriff Selbstorganisation meint dabei, sich eigenständig zu strukturieren und zu ordnen innerhalb verschiedener Prozesse, seien diese nun beruflich gefordert oder privater Natur (Pfister und Müller 2019, S. 40).

Arbeiten im Homeoffice beschreibt nun einen Kontext, in dem berufliche Arbeit im privaten Raum verrichtet wird. Abläufe und ihre Kontrolle, wie sie in einem betrieblichen „Präsenzkontext“ vorzufinden sind, verflüssigen sich. Insbesondere erscheint es notwendig, verschiedene Organisationsprozesse wie den Austausch von Informationen, Möglichkeiten der Entscheidungsfindung und die Bewertung von Arbeitsleistung neu zu definieren und angemessene Umgangsweisen damit zu entwickeln (Laloux 2015, S. 99).

**Figure 1:**
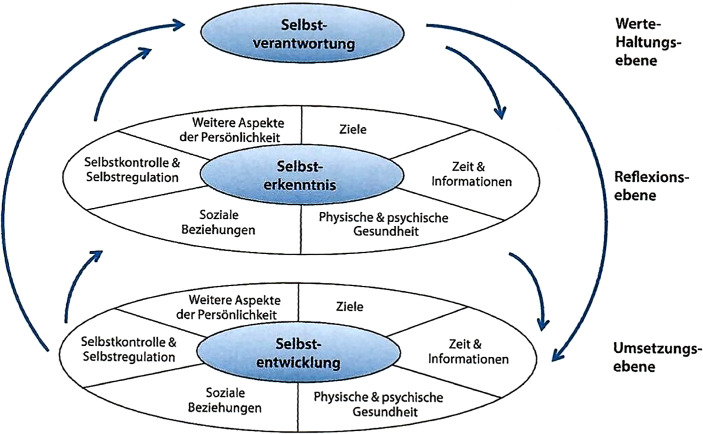

Individuelle Selbstorganisation in beruflichen Prozessen führt dementsprechend auch zu erhöhter Verantwortlichkeit bei den einzelnen Mitarbeiter:innen. Dabei lässt sich Selbstorganisation als Phänomen innerhalb sozialer Systeme beschreiben, dass sich die eigene Ordnung aus den Informationen und Erfahrungen des Systems selbst heraus gestaltet (Altherr 2019, S. 414). Nicht mehr die vorgegebenen Abläufe und Strukturen verantworten die Prozesse, sondern die einzelnen Mitarbeiter:innen übernehmen organisationale Verantwortung. „Selbstmanagement-Kompetenz umfasst die Bereitschaft und die Fähigkeit, das eigene Leben selbstverantwortlich zu steuern und so zu gestalten, dass Leistungsfähigkeit, Leistungsbereitschaft, Wohlbefinden und Balance gestärkt und langfristig erhalten werden. Selbstmanagement ist gelebte Selbstverantwortung“ (Graf 2019, S. 12). Selbstmanagement integriert dabei verschiedene bedeutsame Bausteine, die insbesondere in der Arbeit im Homeoffice Beachtung finden sollten (Abb. 1).

Ausgegangen wird dabei von der Selbstverantwortung als Kern des Modells mit der Anforderung, die eigene Lebensführung und damit auch das berufliche Tun mit persönlichen Werten und Prinzipien abzugleichen und verantwortlich zu gestalten. Die Reflexionsebene fokussiert die Selbsterkenntnis und beinhaltet wesentliche Bausteine der persönlichen Ausstattung und der möglichen Ressourcen. Die Umsetzungsebene als dritte Ebene reflektiert die Selbstentwicklung und mündet idealerweise in konkrete Handlungen (Graf und Olbert-Bock 2019, S. 289). Gerade in dieser Ebene erscheinen angemessene strukturelle Bedingungen nötig, um den Anforderungen der überwiegend individuellen Bausteine auch gerecht werden zu können.

Obwohl selbstorganisierte Arbeit oft dazu führt, dass länger und intensiver gearbeitet wird, wird sie von Beschäftigten grundsätzlich wertgeschätzt. Arbeitnehmer:innen sehen darin Chancen und erweiterte Spielräume, worin auch ein salutogenes Potenzial liegt. Andererseits wirkt dieses subtile Management der Leistungsanforderungen als Motor für selbstausbeuterische Tendenzen. „Wenn Erfolge winken oder Misserfolge drohen, überschreitet man die vertraglich vereinbarten Arbeitszeiten von sich aus – und zwar auch dann, wenn man weiß, dass man damit auf eigene Rechte verzichtet und womöglich die eigene Gesundheit gefährdet“ (Peters 2011, S. 118). Es zeigt sich, dass Mitarbeiter:innen trotz traditioneller Regulierungskriterien (Einhaltung von Pausen und tägliche Höchstgrenzen bei der Arbeitszeit) im Homeoffice bei hohem Leistungsdruck diese weitgehend ignorieren. Es entsteht somit eine „interessierte Selbstgefährdung“, welche zu Einschränkungen der persönlichen lebensweltlichen Interessen der Mitarbeiter:innen führen kann. Dieses Phänomen betrifft vor allem Männer, wodurch insbesondere für sie eine höhere Gefährdung ihrer Gesundheit bei selbstorganisiertem Arbeiten besteht (Ahlers und Lott 2018, S. 18 f.).

Selbstmanagement stellt also eine individuelle Aufgabe für Mitarbeiter:innen dar, die allerdings entsprechende strukturelle Rahmenbedingungen benötigt. Im Homeoffice zählen dazu die räumlich-technische Ausstattung sowie eine kommunikative (betriebliche) Umgebung, in der die Reflexionsanforderungen von Selbstorganisation beachtet und idealerweise gefördert werden. Dies erscheint umso schwieriger, da sich im Homeoffice keine „informellen“ Räume und Zeiten für Reflexion in den verschiedenen Ebenen des Selbstmanagements automatisch ergeben. „Für die betriebliche Gestaltung wird es zukünftig wichtig sein, die jeweiligen Unternehmenskulturen mit ihren Leistungsanforderungen (als Rahmenbedingung der Digitalisierung) aufzudecken, die Verfügbarkeit notwendiger Zeitressourcen der Beschäftigten zu thematisieren und soziale Unterstützung sowie Qualifikationsbedarfe z. B. im Grenzmanagement einzufordern“ (ebd., S. 21; vgl. Rose 2018).

### Kommunikationsprozesse als Herausforderung im Homeoffice

Eine zentrale Herausforderung für Mitarbeiter:innen im Homeoffice scheint die Kommunikation zu sein. Zwar bieten digitale Technologien mittlerweile vielfältige Möglichkeiten, miteinander zu sprechen und sich dabei per Video auch zu sehen, allerdings werden in dieser Form der Kommunikation „Auslassungen“ produziert. Werden digitale und soziale (persönliche) Kommunikationsmodelle miteinander in Bezug gebracht, so zeigt sich rasch ein wunder Punkt: In der digitalen Kommunikation werden wesentliche analoge Informationen nicht mittransportiert, und somit wird die Interpretation des Kommunizierten beim Empfänger deutlich erschwert. Der soziale und der physische Kontext muss also von den Beteiligten eigenständig mitgeneriert werden, da er nicht einfach wie in der analogen Kommunikation zur Verfügung steht (Sweet und Schiermayr 2020, S. 90 f.). Weibler (2021, S. 59–63) beschreibt neben diesen Einschränkungen verständigungsorientierter Kommunikation in digitalen Räumen auch eine Möglichkeit, wie Führungskräfte zu Mitarbeiter:innen mittels eines erlernbaren dialogorientierten Ansatzes einen bereichernden Austausch entwickeln können, und weist damit auf die Notwendigkeit hin, entsprechende Angebote zu entwickeln bzw. umzusetzen.

Aus gesundheitsförderlicher Sicht erscheint es bei zunehmender Digitalisierung und Ausweitung von Homeoffice daher notwendig, partizipative Kommunikationsprozesse bzw. -räume zu initiieren. Ziel ist es, Kommunikation zu unterstützen und die oben beschriebenen „Auslassungen“ zu minimieren. Es könnte daher die Möglichkeit zum Dialog mit unterschiedlichen Mitarbeiter:innen oder Gruppen eingerichtet werden (Pieck et al. 2019, S. 149).

### Vertrauensvolle Verständigung in der digitalen Begegnung

Es zeigt sich, dass bei stark reduziertem Face-to-Face-Kontakt von Mitarbeiter:innen die Gestaltung dieser Kontakte und die Pflege der beruflichen Beziehung unter den Beschäftigten deutlich erschwert sind. Bei Mitarbeiter:innen kann in der Folge die Identifikation mit den betrieblichen Zielen und dem beruflichen Kontext verloren gehen, und die Bereitschaft, sich zu engagieren und dem betrieblichen Umfeld Vertrauen entgegenzubringen, kann sinken (Van Knippenberg und Van Schie 2000, S. 141). Die persönliche Motivation für die berufliche Tätigkeit leidet, wenn das eigene Engagement im beruflichen Kontext nicht erkannt und nicht ausreichend gewürdigt wird (Staar et al. 2019, S. 221). Vertrauensvolle Verständigung entsteht vor allem in jenen Kontexten, in denen Mitarbeiter:innen gemeinsame (positive) Erfahrungen sammeln können. Im Face-to-Face-Kontakt können diese Erfahrungen vielfach ohne spezielle Planung und Organisation sozusagen „nebenbei“ von den Mitarbeiter:innen gemacht werden. Natürlich eignen sich zur Entwicklung von Vertrauen in Teams und Arbeitsgruppen auch verschiedene Trainings, Coaching, Aktivitäten zur Teamentwicklung und gemeinsame Fortbildungen usw., wie sie ja vielfach auch angeboten werden. Niklas Luhmann konstatierte schon vor über 50 Jahren: „Wo es Vertrauen gibt, gibt es mehr Möglichkeiten des Erlebens und Handelns“ (Luhmann 2014, S. 8). Die Arbeit im Homeoffice birgt also das Risiko eines Vertrauensverlusts und damit einhergehend einer Einschränkung von Verständigung und Perspektivenwechsel. Allerdings tragen gerade diese Eigenschaften einen wesentlichen Anteil zur erfolgreichen Gestaltung beruflicher Aufgaben bei und stellen gesundheitsrelevante Aspekte für Mitarbeiter:innen dar.

Ein Auslassen von Feedback und vertrauensvoller Verständigung stellt eine Belastung für die Ausgestaltung der jeweiligen beruflichen Rollen dar und führt zu dem Risiko, in der eigenen Perspektive zu „erstarren“, und zu einem damit einhergehenden Ansteigen psychischer Belastung.

## Forschungsdesign und Umsetzung von Forschung und Entwicklung

Die Forschung und Entwicklung in der Umsetzung des Projekts erfolgte in Kooperation mit vier Betrieben aus unterschiedlichen Branchen: Je ein Unternehmen aus der Metallindustrie, Pharmaindustrie, Unterhaltungsbranche und der Gesundheits- und Sozialbranche waren am Projekt beteiligt. Die Entwicklung dieses Assistenzangebots erfolgt grundsätzlich in Kooperation mit den Mitarbeiter:innen und Betriebsrät:innen der einzelnen Unternehmen. Die Vorgehensweise wurde partizipativ angelegt und band die betroffenen Mitarbeiter:innen und Führungskräfte mit ein. Zu Beginn des Projekts wurden nach einer ersten Literaturanalyse mittels Gruppen- und Einzelinterviews mit den Homeoffice-Mitarbeiter:innen Belastungsbereiche ermittelt. Die Auswertung der Interviews führte zur Identifikation von relevanten Belastungsbereichen als Grundlage zur Entwicklung von Assistenzangeboten.

Der empirische Zugang zum Projekt wurde im Sinne der Interventionsforschung umgesetzt: Sie fokussiert partizipative Prozesse, durch die sich Möglichkeiten ergeben, um offen und praxisorientiert an wesentlichen Themenstellungen zu arbeiten. Durch diesen Zugang können die Prozessbeteiligten selbst bestimmen, welcher Fokus der Auseinandersetzung für sie relevant erscheint, und weitere Entwicklung selbst steuern. Mittels laufender Reflexion des praktischen Tuns fördert die Interventionsforschung eine Weiterentwicklung in der betrieblichen Praxis und unterstützt die Entwicklung neuer Perspektiven (Ukowitz 2016, S. 10 f.).

### Umsetzung des geplanten Designs

Zu Beginn des Projektes wurden unter Berücksichtigung aktueller Forschungserkenntnisse (Hasenbein 2020; Steidelmüller 2020; Ferreira und Vogt 2021) Interviewleitfäden für die Befragung von Mitarbeiter:innen mit Homeofficeerfahrung entwickelt. Diese stellen die Grundlage zur Erfassung bzw. Analyse von Belastungsbereichen von Mitarbeiter:innen dar. Mit den Personalverantwortlichen, Abteilungszuständigen und Geschäftsführungen der vier kooperierenden Betriebe wurde die Umsetzung der Forschungs- und Entwicklungsprojektes koordiniert und die Datenerhebung koordiniert.

Die Interviews konnten nur zum Teil persönlich geführt werden, da manche Betriebe auch als „systemrelevant“ eingestuft sind und daher ein persönlicher Kontakt nicht möglich war. Daher wurden auch Interviews über Videoplattformen geführt. Parallel zu den Interviews erfolgten auch die Auswertung und die Kategorisierung der durch die Gespräche erhobenen Informationen. Auf diesen Grundlagen wurden wesentliche Themenbereiche für die Unterstützungsmodule identifiziert. Aufbauend auf diesen aktuellen Erkenntnissen und der Literaturanalyse zum Themenbereich wurde S.P.A.S.S. (Systemic Personal Assistance Service Solution) konzipiert.

In der Pilotphase dieser ersten Umsetzung wurden die Module immer von zwei Trainer:innen durchgeführt. Damit konnte eine ausreichende inhaltliche Reflexion sichergestellt werden, die in die Weiterentwicklung der Module einfloss. Die am Modul beteiligten Mitarbeiter:innen wurden im Anschluss an die Teilnahme um Statements gebeten, die wiederum ausgewertet wurden und in die inhaltliche und strukturelle Weiterentwicklung des Assistenzprogramms Eingang fanden.

### Formen und Umfang der Datenerhebung

Die explorative Herangehensweise soll vor allem neue Erkenntnisse sowohl auf theoretischer als auch auf Anwendungsebene hervorbringen. Daher sollten solche qualitativen Methoden zur Datengewinnung herangezogen werden, mit denen neue oder unerwartete Befunde generiert werden können und die Möglichkeit für Unerwartetes offengehalten werden kann (Döring und Bortz 2016, S. 192). In erster Linie wurde zur Datenerhebung die Gruppendiskussion eingesetzt. Um Meinungen bzw. Wahrnehmungen besser abzubilden und wiederzugeben, erscheint die Gruppe als Mittel günstiger zu sein. Auch kann eine Gruppe bei vorgegebenen Problemlagen oft kreativer und umfassender zur Entwicklung von neuen und hilfreichen Strategien beitragen (Flick 2012, S. 250 ff.).

Eine weitere Erhebungsmethode im Forschungsprojekt stellte das fokussierte Interview dar, das sich gerade für die vorliegende Fragestellung und das Entwicklungsinteresse anbot. Dieses Interview nützt die Gesprächsanreize einer schon erlebten sozialen Situation und eignet sich insbesondere dafür, persönliche und nicht immer so einfach zu erinnernde Inhalte von Situationen wieder darzustellen (Hopf 2012, S. 353 ff.). Für die Befragungen wurde ein am Projektziel orientierter Leitfaden entwickelt und von den Interviewleiter:innen eingesetzt.

Neben den acht vorbereitenden Gesprächen mit den einzelnen Koordinator:innen bzw. Verantwortlichen für das Projekt in den jeweiligen Betrieben wurden Interviews mit 26 Mitarbeiter:innen und Betriebsrät:innen geführt. Die Gespräche wurden in der Folge transkribiert. Das Kategoriensystem wurde in einem ersten Schritt aus den theoretischen Grundlagen entwickelt und nach der Auswertung der ersten Interviews aufgrund der empirischen Daten noch erweitert bzw. ergänzt. Somit erfolgte die Kategorienbildung in einer kombinierten deduktiven und induktiven Form. Nach Auswertung der gesamten Interviews fanden mit den beteiligten Projektpartner:innen vier Feedback- bzw. Reflexionsgespräche mit Teilnehmer:innen und Leitungspersonen der Pilotworkshops zu den Ergebnissen statt. Diese Vorgehensweise orientierte sich an der „Struktur-Lege-Technik (SLT)“ (Flick 2012, S. 205) und ermöglicht eine Weiterentwicklung und Differenzierung wesentlicher Inhalte. Mittels einer weiteren inhaltsanalytischen Auswertung dieser zusätzliche Reflexionsschleife konnten die Inhalte und die Struktur des Assistenzworkshops weiterentwickelt und neuerlich umgesetzt werden.

### Entwicklung des Kategoriensystems

Die Entwicklung von Kategorien ist stark abhängig von der Fragestellung und/oder vom empirischen Material bzw. von dem Interesse der Forschung, also den Forschungsfragen und dem theoretischen Wissensbereich, der zur Verfügung steht. Bei einem hohen Ausmaß an Vorwissen und Erfahrung mit dem Gegenstand des Interesses lassen sich schon vor der Analyse des erhobenen Datenmaterials erste Kategorien bilden. Um den Inhalten der erhobenen Daten gerecht zu werden, werden häufig in einem zweiten Schritt zusätzliche Kategorien aus dem Material entwickelt bzw. schon bestehende Kategorien verändert. Dies entspricht einer deduktiv-induktiven Kategorienbildung (Kuckartz 2012, S. 59–71).

Schon nach den ersten Gruppendiskussionen wurden die Aufzeichnungen transkribiert und ein Kategoriensystem, aufbauend auf theoretischen Grundlagen, erarbeitet. Die zu diesem Zeitpunkt zur Verfügung stehenden Daten wurden mithilfe vorab deduktiv entwickelter Hauptkategorien analysiert. Aus dieser ersten Auswertung und Analyse ergab sich die Notwendigkeit, verschiedene Themenbereiche zu differenzieren, und in der Forschungsgruppe wurden, ausgehend vom vorliegenden Datenmaterial, relevante weitere Kategorien formuliert. Nach nochmaliger Analyse von mehreren transkribierten Gruppendiskussionen stand das nunmehr vorliegende Kategoriensystem zur inhaltsanalytischen Auswertung zur Verfügung. In dem vorliegenden Entwicklungsprojekt wurden sowohl die Hauptkategorien als auch in weiterer Folge die Unterkategorien in einer Mischform, also deduktiv/induktiv, entwickelt. Dabei wurde im Bereich der Auswirkungen primär auf Belastungen fokussiert und auf eine Darstellung von Beanspruchungsaspekten sowie der Möglichkeit von Kompensation durch Ressourcen verzichtet. Die Übersicht zum Kategoriensystem wird in Tab. 1 dargestellt.

**Table 1:** 

Hauptkategorie	Unterkategorie	Definition
1. Übliche Unterstützungssysteme	Innerbetrieblich	Übliche und bekannte zur Verfügung stehende Unterstützungssysteme bei Unsicherheiten und Überforderung
	Extern	Unterstützungssysteme außerhalb des Unternehmens, die angefragt werden können
	Betriebsrat	Betriebsrat als Unterstützung in unsicheren, herausfordernden betrieblichen Realitäten
2. Kommunikationsstrukturen	Horizontal	Werden Veränderungen in der Kommunikation auf der Kolleg:innenebene erlebt? Findet Kommunikation auf der jeweils gleichen Hierarchieebene statt?
	Vertikal	Wie hat sich die Kommunikation auf unterschiedlichen Hierarchieebenen dargestellt und entwickelt?
	Betriebsrat	Position des Betriebsrats in den innerbetrieblichen Kommunikationsstrukturen
3. Belastungen/Chancen	Im Arbeitsprozess	Homeoffice zwischen Autonomie/Eigenständigkeit und dem Risiko der Einsamkeit/Überforderung
	Privat	Herausforderungen und Vorteile in der privaten Alltagsgestaltung
	Überlappend	Abgrenzungen und Verknüpfungen zwischen beruflich und privat
4. Auswirkungen	Psychisch	Beschreibungen von psychischen Belastungsfaktoren
	Physiologisch	Beschreibungen von physischen Belastungsfaktoren
	Sozial	Beschreibungen von sozialen Belastungsfaktoren
5. Anliegen der Betroffenen	–	Darstellung der wesentlichen Anliegen von Mitarbeiter:innen im Homeoffice

### Auswertung mittels qualitativer Inhaltsanalyse

Die Auswertung des Textmaterials aus den Gruppendiskussionen und den Einzelinterviews wurde mittels der qualitativen Inhaltsanalyse umgesetzt. Dieses Phasenmodell, das aufeinanderfolgende Schritte beschreibt, entwickelt eine Struktur, die es ermöglicht, Informationen bzw. Variablen herauszufiltern. Dabei wird das vorhandene Datenmaterial innerhalb der einzelnen Kategorien komprimiert und reduziert und bei der Codierung entsprechend den Kategorien interpretiert (Kuckartz 2012, S. 34 ff.). Im Zuge dieser zusammenfassenden Analyse wurde für jedes Interview eine Themenmatrix erstellt, um auch die nachfolgende Auswertung und Ergebnisdarstellung zu strukturieren. Im Anschluss an diesen ersten Schritt der Inhaltsanalyse wurden die einzelnen Themenmatritzen wiederum zusammenfassend analysiert und ausgewertet. Nachfolgend werden die wesentlichen Erkenntnisse dargestellt, die in weiterer Folge zur Entwicklung des Unterstützungstools S.P.A.S.S. führten.

## Ergebnisse

Die ausgewerteten Interviews in den oben dargestellten Kategorien führen zu wesentlichen Erkenntnissen zu den Belastungsfaktoren der Mitarbeiter:innen und zu Schlussfolgerungen für die Entwicklung eines Unterstützungstools. So weist die Auswertung darauf hin, dass es insbesondere zu Beginn der Umstellung auf überwiegend Homeofficebetrieb Herausforderungen mit der „technischen Ausstattung“ zu Hause gab, die aber zunehmend weniger wurden.

Es war eine sensationelle Situation eigentlich. Home Office without office halt […] Das Notebook habe ich mit meiner Lebenspartnerin gemeinsam benutzt, die auch HO gehabt hat. Und dann haben wir noch zwei Kinder, und die haben Homeschooling gehabt. […] Für mich war es gewaltig schwierig. Ich habe es genossen eigentlich, wenn ich einmal rauskomme von daheim. (Interview)

Auf der Ebene von Arbeitsorganisation zeigen sich vielfach deutliche, sowohl strukturelle als auch persönliche Herausforderungen. Themen wie Erreichbarkeit, Pausengestaltung, Abstimmung mit Kolleg:innen bei komplexeren Aufgaben und die individuelle Organisation von beruflichen und privaten Aufgaben wurden von den Mitarbeiter:innen als zum Teil sehr belastend identifiziert.

„Mir ist das überhaupt nicht leicht gefallen, dass ich sage: ‚Jetzt ist Arbeitsende‘ und ich hebe nicht mehr ab beim Telefon. Also, weil sich Privat und Arbeit dann so überschneiden, wenn man da nicht ganz strikt ist. Also das ist mir nicht ganz leicht gefallen, zu sagen, jetzt habe ich ausgestempelt und hebe dann nicht mehr dienstlich ab.“ (Interview)

„Aber, weil man quasi alles auf einmal daheim geballt hat … also das war extrem belastend und das hat auch zu viel Streitpotenzial geführt.“ (Interview)

In diesem Spannungsfeld von Selbstorganisation und Strukturunsicherheit lassen sich verschiedenste Belastungsfaktoren bei den Mitarbeiter:innen identifizieren. Dieser Themenbereich weist auf ein Unterstützungsbedürfnis hin, das teilweise auch von Mitarbeiter:innen konkret benannt wurde.

Ein zweiter Themenbereich zu Herausforderung oder Belastung tritt im Bereich von Kommunikation und der damit einhergehenden Verunsicherung hervor. Für Mitarbeiter:innen im Homeoffice wird es offenbar zunehmend schwieriger, zu erkennen, was „richtig ist“ – also welche Vorgehensweisen und Abläufe angemessen erscheinen, da der kollegiale Austausch auf Mitarbeiter:innenebene weniger wurde und die Kommunikation sich eher auf die jeweiligen Vorgesetzten konzentrierte. Mit dieser Einschränkung von Feedback geht auch eine Verunsicherung hinsichtlich des eigenen „Arbeitserfolgs“ einher und damit eine Verringerung von Arbeitszufriedenheit.

Also wenn man gerade mal eine Idee hat, dann teilt man sich das ja mit. Das tut man nicht, wenn man zu Hause ist. Man schreibt es vielleicht auf, sammelt seine Sachen dann wieder für eine Besprechung. Es wird alles so megaeffizient, was auch ganz schön ist, aber ich glaube nur, das geht nicht. Also, weil dann die ganze Zwischenmusik oder die Zwischentöne halt einfach fehlen oder vielleicht eben die Zeit, um irgendetwas zu verarbeiten. (Interview)

Es zeichnen sich darüber hinaus noch weitere Themenbereiche ab, die Herausforderungen für Mitarbeiter:innen beschreiben und in der Gestaltung von Assistenzprogrammen Berücksichtigung finden können. An dieser Stelle soll allerdings auch auf einzelne Unterstützungssysteme hingewiesen werden, die von befragten Mitarbeiter:innen als hilfreich identifiziert worden sind. In einigen Unternehmen würde ein Unterstützungstool durchaus begrüßt werden, erste Ansätze dazu stehen für Mitarbeiter:innen ohnehin schon zur Verfügung.

Weil intern, wenn du dich austauschst, da ist dir schon viel geholfen, und ein bisschen flexibler ist man dann auch, weil der eine denkt so, der andere so, und dann ist das ein besserer Lösungsansatz. Extern? Ja, wenn man sich von Zeit zu Zeit mal darüber unterhalten kann … wie man jetzt Kundenkontakt, oder so… wie wir das jetzt eh schon haben. Das ist eh super. (Interview)

Aus dieser Auswertung der Daten aus den Gruppendiskussionen und Interviews ergaben sich drei Themen für das Assistenzprogramm, die zentrale Belastungsbereiche adressieren und in wesentlichen Inhalten auch mit den theoretischen Erkenntnissen der Literaturanalyse korrelieren:

1. Wie gelingt meine persönliche Selbstorganisation im Homeoffice?
2. Feedback und Kommunikation – ich leiste gute Arbeit!
3. Ich gehöre dazu – Verständigung und Perspektivenwechsel!

Die inhaltliche und methodische Erarbeitung der Module führte zur Konzeption von S.P.A.S.S. (**S**ystemic **P**ersonal **A**ssistance **S**ervice **S**olution), das mittlerweile schon in den Betrieben Umsetzung fand. Nachfolgend soll S.P.A.S.S. als einfach umzusetzende und von den Mitarbeiter:innen und Betriebsrät:innen als hilfreich angesehene Unterstützung in Zeiten von Homeoffice vorgestellt werden.

## Systemic Personal Assistance Service Solution – S.P.A.S.S.

Als lösungsorientiertes systemisches Tool möchten die Autor:innen das Konzept „S.P.A.S.S. (Systemic Personal Assistance Service Solution) – Workshop“ anbieten. Die lösungsorientierte systemische Herangehensweise orientiert sich dabei an der von de Shazer (2002) beschriebenen Haltung, Prozesse lösungsorientiert und unvoreingenommenen zu begleiten. Dieser Zugang entspricht auch dem systemtheoretischen Grundkonzept von Luhmann (2006, S. 92ff.), das die Autopoiese und somit die operationale Geschlossenheit sozialer Systeme erkennt. Er beschreibt, dass soziale Systeme nur offen sind für Kommunikation und Information, und somit können sie durch Kommunikation in einer Weise gestört bzw. irritiert werden, die zur Neuorganisation bewegt. Dieses Tool präsentiert sich als Dialogveranstaltung zwischen unterschiedlichsten Akteur:innen eines Unternehmens, die in der intensiven, eher einseitig eingeleiteten Homeoffice-Phase wenig Möglichkeit zum informellen Austausch hatten.

Das Workshop-Programm orientiert sich an den Erkenntnissen aus der Erhebungsphase und unterscheidet vier Themenschwerpunkte mit jeweils angepassten methodischen Zugängen und Übungen. Die Schwerpunkte „Selbstführung“, „Orientierung“, „In Kontakt kommen und sein“ sowie „Kommunikation und Kontext“ erlauben es den Teilnehmenden, sich selbst mit Blick auf ihre grundsätzliche Persönlichkeitsstruktur – also die individuellen psychischen und physischen Merkmale und die charakteristischen Anpassungsweisen an sich verändernde Gegebenheiten – und ihr Verhalten im Homeoffice zu reflektieren und auszutauschen.

Der persönliche Lebensrahmen der Einzelnen spielte eine entscheidende Rolle hinsichtlich des möglichen oder verunmöglichten Umgangs mit räumlichen, technischen, inhaltlichen oder höchstpersönlichen Herausforderungen. Beispielsweise kamen Eltern von kleinen Kindern, die möglicherweise auch noch auf relativ engem Raum zusammenlebten und beide berufstätig waren, schneller in Konfliktsituationen als Alleinlebende, die wiederum schnell von Einsamkeitsgefühlen heimgesucht wurden. Fragen der Öffnung und Abgrenzung, des Privaten und des Öffentlichen, der Erreichbarkeit und des Abtauchens stellten sich allerorts, und die Kommunikation wurde im digitalen Bereich vertikaler angesetzt als zuvor, d. h. der informelle Austausch auf der horizontalen Hierarchieebene kam teils zum Erliegen bzw. es wurde ihm keine Plattform geboten, wenn die Mitarbeitenden nicht selbst die Initiative dazu ergriffen.

**Figure 2:**
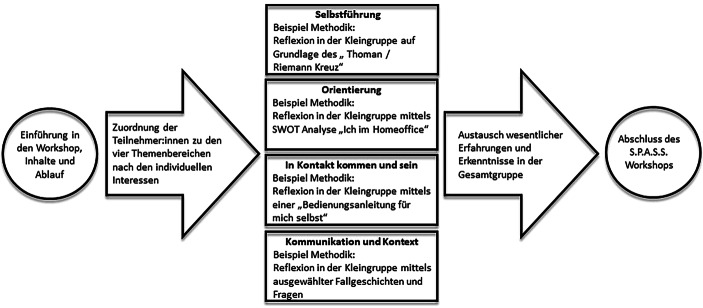

Der S.P.A.S.S.-Workshop ist konzipiert, um die Mitarbeitenden eines Unternehmens auf der informellen Ebene zum Reflektieren und zum Kommunizieren zu bringen, sodass extrinsische und intrinsische Prozesse – wie z. B. der Dialog zwischen allen Stakeholdern und die Reflexion der eigenen Persönlichkeitsstruktur und des eigenen kontextbezogenen Verhaltens – in Gang gesetzt und unterstützt werden im Sinne einer systemischen Intervention, einer „Irritation“ (Krause 2005, S. 169), die neue individuelle und umweltbezogene Selbstorganisationsprozesse in Gang setzt. Der Workshop sollte von Externen moderiert werden und kann sowohl in Präsenz als auch digital abgehalten werden. In der Testphase wurde z. B. die Plattform „wonder.me“ genutzt, die es den Teilnehmenden erlaubt, sich in Gruppen zusammenzufinden und gleichzeitig den gesamten Teilnehmer:innenraum im Blick zu behalten. Sehr selbstbestimmt können Teilnehmende somit steuern, an welchen Diskussionen sie sich beteiligen wollen und mit wem sie in Kontakt treten möchten. Diese Selbstbestimmung, im Gegensatz zur digital-hierarchischen Rollenzuschreibung, die bei der Homeoffice-Kommunikation im Vordergrund zu stehen scheint, ist ein Grundpfeiler des Workshops. S.P.A.S.S. erlaubt es Arbeitgeber:innen bzw. Arbeitnehmer:innenvertretungen, eine Dialogveranstaltung anzubieten, die Kommunikationskanäle öffnet und zugleich die eigene Rolle der jeweiligen Kommunizierenden reflektiert. Wenn Spannungen zu Tage treten, werden sie durch die Selbstreflexion bereits moderiert und können gemeinsam betrachtet und aufgearbeitet werden. Erfahrungen können geteilt und verglichen werden, wodurch sich für manche neue Perspektiven auftun bzw. Einblicke in andere Lebenswelten gewährt werden. Zudem ist es möglich, konsensierend zu arbeiten, d. h. Vorschläge auf möglichen Widerspruch bei Beteiligten zu überprüfen und somit die bisher möglicherweise nicht gehörten (weil nicht angeforderten) Widerstände zu erkennen, einzuordnen und zu bearbeiten (vgl. Abb. 2).

### Präsenzworkshop S.P.A.S.S.

Die ideale Teilnehmer:innenzahl für den Workshop bewegt sich zwischen 8 und 12 Personen. Auf diese Weise ist ein Austausch auch in wechselnden Kleingruppen sinnvoll möglich. Als Zeitrahmen für den Workshop wurde in der Umsetzungsphase 90 min gewählt. Dies erwies sich als angemessener Zeitrahmen, wobei nicht immer alle Teile des Workshops umgesetzt werden konnten. Allerdings hatten die Teilnehmenden grundsätzlich die Freiheit, je nach Interesse auszuwählen, mit welchem Themenangebot sie mit den Kolleg:innen in Dialog treten wollen.

Moderiert wurde S.P.A.S.S. von jeweils zwei Mitarbeiter:innen des Projekts, um auch die Reflexion der Umsetzung sicherstellen zu können. Es zeigte sich eine hohe Akzeptanz von S.P.A.S.S., die dazu führt, dass dieses Angebot schon jetzt nach der Pilotphase in zwei der vier Unternehmen weitergeführt wird, zum Teil mit externer Unterstützung und zum Teil mit internen Ressourcen. Zur Umsetzung von S.P.A.S.S. wurde eine kurze Power Point Präsentationmit den wesentlichen Inhalten und Fragestellungen/Übungen erstellt. Diese Fragen und Übungen können rasch und problemlos erweitert oder umgestellt werden, damit der Workshop je nach Bedarf und Anforderung adaptiert werden kann. Somit ist S.P.A.S.S. kein eingeschränktes Tool für lediglich wenige Themen oder Belastungsbereiche, sondern stellt ein flexibel einsetzbares Dialogangebot dar.

### Onlineworkshop S.P.A.S.S.

In der Onlineversion des Workshops wurden prinzipiell dieselben Materialen verwendet wie in der Präsenzform. Dabei wurde auf die Konferenz-Plattform „wonder“ zurückgegriffen. Diese Plattform erlaubt den Teilnehmenden ein hohes Maß an Eigenständigkeit und Spontaneität. Sie navigieren darin selbstständig und können selbstgewählt in Dialog treten. Die einzige Herausforderung für manche Teilnehmenden war, dass die Plattform nur englischsprachig zur Verfügung steht und beim Einstieg die Hinweise daher nur auf Englisch beschrieben wurden. Die Anwendung auch für den Onlineworkshop ist ebenso wie die Präsenzversion ohne besondere Fortbildung möglich. Daher eignet sich S.P.A.S.S. als „niederschwelliges Tool“ insbesondere als Anregung zum Dialog innerhalb von Arbeitsgruppen oder individuell initiierte Gruppen in Betrieben.

## Zusammenfassung und Ausblick

Das vorliegende Forschungs- und Entwicklungsprojekt betrachtete die Veränderungen der Anforderungen an Mitarbeiter:innen durch die zunehmende Digitalisierung in beruflichen Kommunikations- und Austauschprozessen. Diese Entwicklung wurde zusätzlich durch die sogenannte „Corona Krise“ beschleunigt. Es zeigt sich, dass die Digitalisierung moderner Funktionssysteme für Gesellschaften eine Zunahme an Flexibilisierung bedeutet. Für alle Beteiligten hat dies Vor- und Nachteile. Auf der Basis relevanter theoretischer Erkenntnisse wurde einerseits erhoben, ob die bekannten neuen Belastungsfaktoren tatsächlich mit jenen korrelieren, die Mitarbeitende in der Krisenphase kolportieren, oder ob sich durch qualitative Befragungen zusätzliche Problemfaktoren identifizieren lassen.

Durch die qualitative Studie im Projekt konnten drei zentrale Themenbereiche identifiziert werden, für die es auch in verschiedenen aktuellen Studien Belege erscheinen (Bachmann und Quispe Bravo 2021; Weibler 2021). Für das zu entwickelnde Assistenzprogramm stellten diese Themenbereiche die Grundlage für die Gestaltung dar:

1. Wie gelingt meine persönliche Selbstorganisation im Homeoffice?
2. Feedback und Kommunikation – ich leiste gute Arbeit!
3. Ich gehöre dazu – Verständigung und Perspektivenwechsel!

Das daraus entwickelte Workshop-Programm S.P.A.S.S. setzt dort an, wo die Limitierung der Digitalisierung offenbart: beim informellen Austausch und Dialog außerhalb hierarchischer Gegebenheiten. In der Gestaltung des Programms wurde insbesondere darauf Wert gelegt, dass es „niederschwellig“ anwendbar ist – also ohne großen zeitlichen und organisatorischen bzw. personellen Aufwand –, um eben den notwendigen Dialog zwischen Mitarbeiter:innen auch in Zeiten von Homeoffice aufrecht zu erhalten. Als Projekt im Sinne der Interventionsforschung entstand innerhalb der vier Betriebe zusätzliche Sensibilität für die Belastungen von Mitarbeiter:innen im Homeoffice, und es wurden Angebote wie S.P.A.S.S. für Mitarbeiter:innen und Teams zur Unterstützung eingeführt.

Das vorliegende Forschungs- und Entwicklungsprojekt legte den Fokus in erster Linie auf Belastungsfaktoren und Beanspruchungen von Mitarbeiter:innen. Allerdings zeigt sich, dass die Arbeit im Homeoffice nicht nur eine Belastung darstellt, sondern unter bestimmten Umständen auch Vorteile und Entlastung für Beschäftigte mit sich bringt. Die Betrachtung von individuellen und strukturellen Ressourcen und ihr Einfluss auf die Belastungsfolgen wurden im vorliegenden Projekt nicht explizit berücksichtigt. Aus den Ergebnissen könnte allerdings abgeleitet werden, dass eine Normierung der Einschätzung von Belastungen und Ressourcen der zunehmenden Individualisierung von Lebens- und Arbeitsbedingungen nur begrenzt Rechnung tragen kann (Ferreira und Vogt 2021). In der Organisationsberatungs- und Coachingpraxis könnten daher Herangehensweisen im Sinne der Interventionsforschung eine Erweiterung darstellen, die dem aktuellen gesellschaftlichen Zeitgeist Rechnung trägt. Oder wie Bauman (2018, S. 187) es ausdrückt: „Wir leben in einer Epoche der Brüche und Diskrepanzen, einer Epoche, in der alles – oder fast alles – möglich ist, während man nichts – oder so gut wie nichts – in der Gewissheit, es zu durchschauen, selbstbewusst angehen kann.“
